# Acupoint Embedding of Polyglactin 910 Sutures in Patients with Chronic Pain due to Cervical Spondylotic Radiculopathy: A Multicenter, Randomized, Controlled Clinical Trial

**DOI:** 10.1155/2018/3465897

**Published:** 2018-09-26

**Authors:** Yu-Xia Chu, Wen-Qiang Cui, Fei Xu, Yuan-Yuan Pan, Yan-Hua Ma, Teng Chen, Si-Jing Wang, Hong-Bo Sun, Yan-Qing Wang, Wen-Shan Sun

**Affiliations:** ^1^Department of Integrative Medicine and Neurobiology, School of Basic Medical Sciences, Fudan University, Shanghai, China; ^2^Institutes of Brain Science, Brain Science Collaborative Innovation Center, State Key Laboratory of Medical Neurobiology, Fudan University, Shanghai, China; ^3^The Institute of Integrative Medicine, Fudan University, Shanghai, China; ^4^Department of Integrative Medicine, Huashan Hospital, Fudan University, Shanghai, China; ^5^The Community Health Service Center in Meilong, Shanghai, China; ^6^Department of Acupuncture, The Second People's Hospital of Shanghai, China; ^7^Department of Traditional Chinese Medicine, The Fifth People's Hospital of Shanghai, Fudan University, Shanghai, China; ^8^The Community Health Service Center in Wujing, Shanghai, China

## Abstract

**Objective:**

We aimed to investigate the effectiveness of acupoint polyglactin 910 (PGLA) embedding in patients with cervical spondylotic radiculopathy (CSR).

**Methods:**

A total of 102 CSR patients with neck and shoulder pain were recruited and assigned randomly into three groups: the sham acupoint embedding (SAE) group, the middle-layer acupoint PGLA embedding (MAPE) group, and the deep-layer acupoint PGLA embedding (DAPE) group. The primary outcomes were Visual Analog Scale (VAS) scores showing the analgesic effects of treatment. Secondary outcomes included clinical symptoms (evaluated by the Yasuhisa Tanaka 20 (YT-20) score and the neck disability index (NDI)) and patient health status (evaluated by the 36-item short-form survey (SF-36)) as reported in the trial.

**Results:**

Compared with the SAE group, VAS scores were significantly reduced at 1, 2, 3, 4, and 10 weeks after the first treatment in both the DAPE and MAPE groups (*P* < 0.001). Moreover, there were statistically significant increases in the weekly YT-20 scores and significant reductions of the weekly NDI scores compared with baseline values in both the DAPE and MAPE groups (*P* < 0.001). Compared with baseline values, both the physical component summary (PCS) and the mental component summary scores of the SF-36 at 2, 3, 4, and 10 weeks were significantly higher in the DAPE and MAPE groups (*P* < 0.001). There were significant lower VAS scores (*P* < 0.01), higher PCS scores (*P* < 0.05) at 3 weeks, and lower NDI scores (*P* < 0.05) at 4 weeks in the DAPE group compared with the MAPE group.

**Conclusions:**

Both DAPE and MAPE showed significant and long-lasting effects on alleviating pain and improving clinical symptoms as well as quality of life in CSR patients with neck and shoulder pain. A more intense effect was seen in the DAPE group compared with the MAPE group.

## 1. Introduction

Cervical spondylotic radiculopathy (CSR) is a neurologic condition characterized by dysfunction of a cervical spinal nerve, the roots of the nerve, or both [1, 2]. In the setting of CSR, because the nerve root of a spinal nerve is compressed or otherwise impaired, the pain and symptomatology can spread far from the neck and can radiate to the arm, chest, upper back, and/or shoulders, which causes significant disability [3]. The chronic pain (pain that lasts for more than 3–6 months) [4] induced by CSR seriously affects patients' quality of life. CSR may be treated conservatively or surgically [5], but because of the associated risks, surgery should only be considered when conservative management has failed [6].

Acupoint stimulation, including the widely known electroacupuncture (EA) and manual acupuncture, is one of the effective conservative therapies for CSR treatment, especially the associated neck and shoulder pain [7, 8]. However, in order to achieve the long-lasting analgesic effects of EA and manual acupuncture in the treatment of CSR and other sources of chronic pain, these techniques need to be applied at high frequency (for example, once per day) [9], which means more suffering for the patients. Low-frequency application (once or twice a week) might be the reason for the lack of effectiveness for EA seen in previous studies for the treatment of chronic pain and other diseases [10–12].

Acupoint embedding (AE) of absorbable material, another traditional acupoint-stimulating alternative therapy widely used in China and other Asian countries, involves the injection of catgut or other absorbable material at the acupoint once a week to treat pain and other disease states [9–11]. AE artificially creates a mild inflammation and activates the interaction of the immune system with the neuroendocrine system [12]. Our group has shown that acupoint catgut embedding in the neck has significant therapeutic effects for CSR [9, 10]. However, catgut induces a variety of immune responses, including allergic reactions and subcutaneous nodules [13]. It is thus worthwhile to develop a less immunogenic material for use in AE and to design rigorous clinical trials to evaluate the effects of AE for CSR treatment. Polyglactin 910 (PGLA), a copolymer formed by polymerizing together nine parts of polyglycolic acid (PGA) with one part of polylactic acid in the presence of a suitable catalyst, has been reported to elicit minimal tissue response [14, 15].

This study aimed to determine whether AE with the new PGLA material has analgesic and therapeutic effects on treating pain and other symptoms of CSR through a multicenter, randomized, controlled clinical trial. We also explored the therapeutic effectiveness of AE over time and the optimal implanted depth of the PGLA sutures.

## 2. Methods

The trial was registered prior to patient enrollment in the CTR platform (ChiCTR-IOR-17013298, Principal investigator: Wen-Shan Sun, date of registration: 11/08/2017.

### 2.1. Study Design

This clinical trial was a multicenter, parallel group, randomized, and controlled study. We recruited patients with CSR and with neck and shoulder pain as the main complaint from the outpatient unit of the Departments of Traditional Medicine in four Chinese clinical centers, the Fifth People's Hospital of Shanghai, Fudan University, the Second People's Hospital of Shanghai, the Community Health Service Center in Wujing, and the Community Health Service Center in Meilong.

### 2.2. Study Population and Protocol

Patients were enrolled in the study from December 2017 to March 2018. The study population consisted of individuals aged 18 to 65 years with CSR. The diagnostic criteria of CSR are referred to in the Guidelines for the Diagnosis and Treatment of Cervical Spondylosis (2011 edition) promulgated by the Chinese Association of Rehabilitation Medicine's cervical spondylosis branch. The symptoms and signs include syndromes of pain and numbness distributed along the spinal nerve roots and having positive intervertebral foramen extrusion and/or brachial plexus pull tests. Moreover, the clinical manifestations and imaging were consistent with the clinical syndromes. Patients with concurrent headaches, nonradiative pain in the upper extremities, and low back pain were not excluded, but neck pain had to be the main symptom. To ensure the identification of all eligible patients, radiology records were audited. Patients in all treatment groups were allowed to use painkillers when recommended by physicians and when necessary (VAS > 7).

The exclusion criteria were history, signs, or symptoms suggesting a potential nonbenign cause for the patient's pain (including previous neck surgery); any evidence of a specific pathological condition such as malignancy, neurological disease, fracture, herniated disc, or systemic rheumatic disease; clinical signs of spinal cord compression or previous neck trauma; obvious vertigo; being pregnant or lactating; currently participating in another clinical trial; hepatic, renal, hematopoietic, endocrine, cardiovascular, or nervous system diseases; tuberculosis; vertebral deformities; mental illness; or having been treated with physical therapy or manipulation therapy for neck pain during the previous 2 weeks.

### 2.3. Physicians

AE was performed by physicians trained in muscular-skeletal problems. The physicians were required to fulfill the following criteria: ≥3 years of experience with AE and participation in the study training sessions about the trial methods, the interventions being tested, and the standards for performing clinical trials (ICH-GCP). Four physicians in four outpatient units in China participated in this study. In order to maximize standardization, all physicians were given onsite training and were provided with an instruction manual and video.

### 2.4. Randomization and Blinding

A total of 102 eligible patients were recruited and were randomly assigned to receive sham acupoint embedding (SAE, n = 34) treatment, middle-layer acupoint PGLA embedding (MAPE, n = 34), or deep-layer acupoint PGLA embedding (DAPE, n = 34) using a computer-generated randomization schedule produced by an experienced statistician. The sealed and sequentially numbered envelopes containing the randomization were opened immediately after baseline assessment. The participants in the SAE, MAPE, and DAPE groups were blinded to treatment. The practitioners could not be blinded to the treatment assignments given the nature of the interventions, but the outcome assessors, data collectors, and statisticians were all blinded to the treatment allocation.

### 2.5. Interventions

Four acupoints were used per treatment. All patients received acupuncture at two obligatory points, including Jiaji (EX-B 2) of C5 and C6 on the affected side and Dazhui (GV14). The other two points were chosen according to the syndrome differentiation of the meridians in the pain region. The potential acupoints included SJ5, GB34, BL60, SI3, LI4, ST44, LR3, and GB40 [16]. The use of additional acupoints other than the prescribed ones was not allowed. We chose the prescriptions after a systematic review of ancient and modern literature [17], consensus meetings with clinical experts, and experience from our previous study [18].

**Figure 1:**
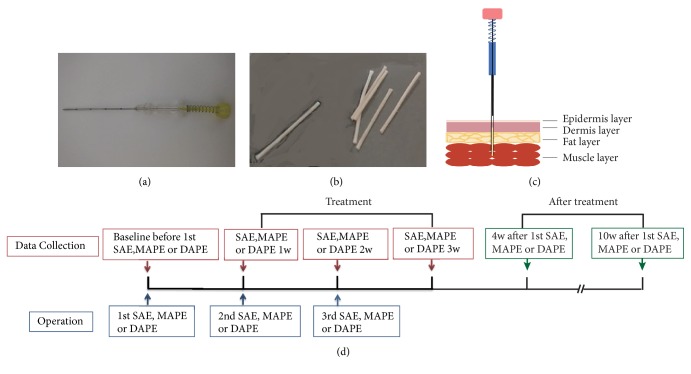
Diagram for acupoint embedding instruments, operations, and data collection. (a) Acupoint embedding needle. (b) PGLA sutures. (c) Schematic of the acupoint embedding of the PGLA. (d) Data collection and operation flow. SAE, sham acupoint embedding group; MAPE, middle-layer (the* semispinalis capitis* muscle layer) PGLA embedding group; DAPE, deep-layer (the* multifidus* muscle layer) PGLA embedding group; 1w, 1 week; 2w, 2 weeks; 3w, 3 weeks; 4w, 4 weeks; 10w, 10 weeks.

For DAPE and MAPE, a disposable stainless embedding needle (diameter 0.03 inches) was used to inject PGLA sutures (0.4 inches in length) into the acupoints (Figure 1). Under the guidance of ultrasound, the PGLA was implanted into the* semispinalis capitis* muscle layer for MAPE and into the* multifidus* muscle layer for DAPE. The sham embedding was also performed under the guidance of ultrasound. The number of needles, the duration of treatment, and the acupoints in the SAE group were identical to those of the MAPE and DAPE groups except that no PGLA was injected. According to our preliminary observation, the analgesic effects of sham embedding into the middle-layer or deep-layer showed no significant difference. Therefore, to minimize the number of patients receiving sham embedding, the embedding needle was inserted into the deep-layer (the* multifidus* muscle layer) in the SAE group. The treatment was given once a week for three consecutive weeks in the three groups. The symptoms and physical signs were observed before and after treatment.

### 2.6. Data Collection

All patients were instructed to complete the CSR case report form, including the VAS of the Short-Form McGill Pain Questionnaire, the Yasuhisa Tanaka 20 (YT-20) score, the neck disability index (NDI), and the physical component summary (PCS) and the mental component summary (MCS) of the 36-item short-form survey (SF-36). All measures were assessed at baseline (1st visit); after 1 week (2nd visit), 2 weeks (3rd visit), and 3 weeks (4th visit) of treatment; at 4 weeks after the 1st visit (1 week after the last treatment); and at 10 weeks after the 1st visit (7 weeks after the last treatment) (Figure 1).

### 2.7. Outcome Measures

#### 2.7.1. VAS Score

The primary outcome was the VAS score, a parameter showing participant-rated pain. The VAS is a 10-cm line with pain descriptors marked “no pain” on the left (scored as 0) and “the worst pain imaginable” (scored as 10) on the right. The patients were asked to report their perceived pain level by marking the VAS with a perpendicular line.

Secondary outcome measures were parameters showing participant-rated disability as follows.

#### 2.7.2. YT-20 Score

The 20-point scale of CSR developed by Yasuhisa Tanaka was adopted to score the clinical symptoms in the patients before and after treatment and to assess the treatment efficacy between groups. A higher score indicates more improved clinical symptoms.

#### 2.7.3. Neck Disability Index

As a measure of neck-specific functional disability, a translated version of the original 10-item NDI was used [19]. The NDI covers 10 dimensions of neck-specific disability, namely, pain intensity, personal care, lifting, reading, headache, concentration, working, driving, sleeping, and recreation. Each item assesses one dimension and is measured on a 6-point scale from 0 (no disability) to 5 (full disability). The overall score is obtained by adding the score for each item. A higher score indicates greater pain and disability.

#### 2.7.4. Assessment of Patient Health Status

Health-related quality of life was assessed using the SF-36, which is a self-reported and generic questionnaire consisting of the following eight domains: general health, bodily pain, physical function, role limitations (physical), mental health, vitality, social function, and role limitations (emotional). The eight domains can be combined into physical and mental sum scales that reflect physical and mental health, and these were the PCS and MCS used in this study. Regression analyses were performed to impute missing values in accordance with the instruction of the developer of the questionnaire [20]. The SF-36 scales were scored according to published scoring procedures, and each scale was expressed using values from 0 to 100, with 100 representing excellent health. This questionnaire was completed at all of the measurement points (baseline and after 1 week, 2 weeks, and 3 weeks of treatment and at the follow-ups 4 weeks and 10 weeks after the first treatment).

### 2.8. Safety

To determine if there are long-term side effects of acupoint PGLA embedding (APE), we performed follow-up observations until 10 weeks after the first treatment. Safety was assessed by spontaneous reporting of adverse effects. We classified serious adverse effects as events that caused death were life-threatening or necessitated admission to hospital.

### 2.9. Quality Assurance

The study had appointed three trained quality inspectors to guarantee the quality of the whole trial. The three inspectors visited each center regularly without prior notice. All patients were telephoned and asked about some details of the trial such as the informed consent, the use of the CSR case report form, and the quality of the treatments in order to judge the normalization of the trial.

### 2.10. Sample Size Calculation

The primary outcome was the VAS scores. With a type I error of 5% and power of 90%, this study required 29 patients per group if the mean decrease in VAS scores after treatment in the SAE, MAPE, and DAPE groups were assumed to be 1, 3, and 2, respectively, and the standard deviations were assumed to be 1.5, 2.1, and 2.5, respectively, if a two-tailed test were used. The sample size was calculated using the PASS software (version 11; NCSS). To account for a possible study drop-out rate of 15%, 102 patients were ultimately enrolled in the present study.

### 2.11. Statistical Analysis

Continuous variables are presented as the mean ± SD with 95% confidence intervals (CIs). The significance level used for the statistical analysis with 2-tailed testing was 5%. The values of the last treatment (3 weeks after APE) were used as a substitute for missing data (values for 4 weeks and 10 weeks in the follow-up period). When comparing the differences within each group at different time points, we used one-way repeated measure (RM) ANOVA. When comparing the differences at different time points among the SAE, MAPE, and DAPE groups, we used two-way RM ANOVA followed by the Tukey multiple comparison test. When comparing the age and duration of illness for these three groups, we used ordinary one-way ANOVA, while the *χ*^2^ test was used for gender difference comparisons. All data in this trial were analyzed using the GraphPad Prism 6 statistical software (version 6.0c; GraphPad Software, Inc.).

This manuscript adheres to the applicable CONSORT guidelines.

## 3. Results

### 3.1. Participants and Baseline Characteristics

**Figure 2:**
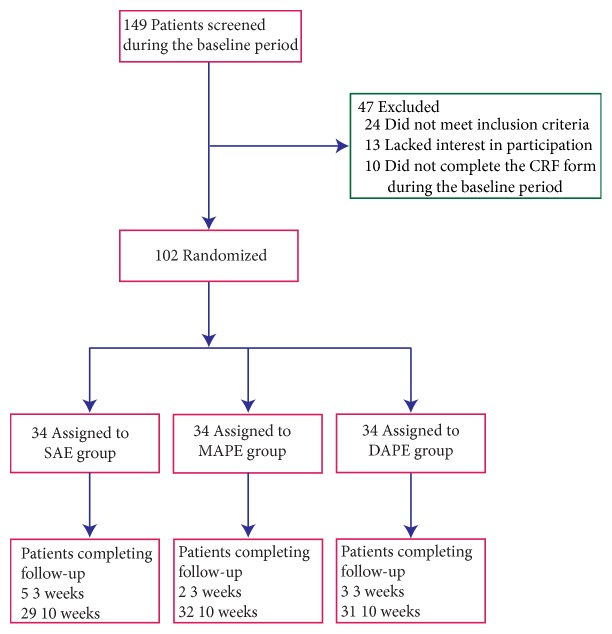
Flowchart of the study protocol according to the CONSORT 2010 statement. SAE, sham acupoint embedding group; MAPE, middle-layer (the* semispinalis capitis* muscle layer) PGLA embedding group; DAPE, deep-layer (the* multifidus* muscle layer) PGLA embedding group.

After the screening of 149 patients, 102 participants 18 to 65 years old were included in the intention-to-treat population and randomized. Ten patients (9.8%) were unable to undergo follow-up (5 in the SAE group, 2 in the MAPE group and 3 in the DAPE group). A total of 92 patients (29 in the SAE group, 32 in the MAPE group, and 31 in the DAPE group) completed the study (Figure 2). The demographic characteristics, including gender, age, and duration of illness, are shown in Table 1, and no significant differences in these characteristics were seen in the three groups (*P* = 0.9, 0.6 and 0.4 for gender, age and duration of illness, respectively).

### 3.2. Primary Outcome

#### 3.2.1. APE Significantly Attenuated Pain, and the Best Analgesic Time Point Was after 3 Weeks of Treatment

The primary outcomes were the VAS scores showing the analgesic effects of treatment. In the DAPE group, there was a statistically significant reduction of the VAS score after 1 week, 2 weeks, and 3 weeks of treatment compared with the baseline values (*P* < 0.001 for all). There were no statistically significant differences in the SAE group at any time point during or after the treatment (*P* = 0.109) (Table 2). In the DAPE group, the mean VAS scores were the lowest after 3 weeks of treatment, indicating that 3 weeks showed the best analgesic effects during the whole treatment period. Moreover, in the DAPE group, the mean VAS scores were still very low at 10 weeks after the first treatment (follow-up period) and significantly lower than SAE group (95% CI, −4.4 to −3.2,* P* < 0.001), indicating a long-lasting analgesic effects of DAPE.

#### 3.2.2. DAPE Had Better Analgesic Effects Than MAPE

It has been proposed that injecting the suture into different tissue layers might influence the analgesic effect of AE; thus we performed DAPE in the* multifidus* muscle layer and MAPE in the* semispinalis capitis* muscle layer and compared the analgesic effects. Similar to the DAPE group, the VAS scores in the MAPE group were significantly decreased compared with baseline (*P* < 0.001). Compared with the SAE group, the VAS scores in the MAPE group were significantly lower at 1 week (*P* < 0.001), 2 weeks (*P* < 0.001), and 3 weeks (95% CI, −4.0 to −2.7,* P* < 0.001) of treatment and at 4 weeks (*P* < 0.001) and 10 weeks (95% CI, −4.4 to −3.2,* P* < 0.001) after the first treatment (follow-up period), while no significant differences were seen at baseline (*P* > 0.05). The VAS scores showed no significant difference between DAPE and MAPE at 1 week or 2 weeks, but they were significantly lower in the DAPE group at 3 weeks after treatment compared with the MAPE group (95% CI, −1.5 to −0.3,* P* < 0.01), indicating more potent analgesic efficacy in the DAPE group (Table 2).

### 3.3. Secondary Outcomes

Secondary outcomes included clinical symptoms (evaluated by the YT-20), a composite of functional status (measured by the NDI), and quality of life assessment (evaluated by the SF-36 health survey) as reported in the trial.

#### 3.3.1. Both DAPE and MAPE Significantly Improved Clinical Symptoms and Function

To test the treatment efficacy of DAPE and other interventions, we measured the changes in YT-20 score and NDI, which are shown in Table 3.

In the DAPE and MAPE groups, there were statistically significant increases in the weekly YT-20 scores after 1, 2, and 3 weeks of treatment compared with baseline values (*P* < 0.001 for both groups). Compared with the SAE group, the YT-20 scores were significantly higher in both the DAPE (95% CI, 2.2 to 6.1,* P* < 0.001) and MAPE (95% CI, 2.3 to 6.3,* P* < 0.001) groups at 3 weeks after treatment. Compared with the SAE group, there were no significant differences at 10 weeks after the first treatment (follow-up period) in the DAPE group (95% CI, −1.4 to 2.2,* P* > 0.05), indicating that the improvement in clinical symptoms by DAPE had disappeared.

In the DAPE and MAPE groups, there was a statistically significant reduction of the weekly NDI score after 1, 2, and 3 weeks of treatment compared with baseline values. Compared with the SAE group, the NDI scores were significantly lower in the DAPE group (95% CI, −11.9 to −5.0,* P* < 0.001) at 3 weeks after treatment. The NDI scores at 4 weeks and 10 weeks after the first treatment (follow-up period) in the DAPE group were significantly higher than baseline values (*P* < 0.001 for both), although still significantly lower than the SAE group (*P* < 0.001 for both), indicating a short-term improvement of neck disability by DAPE. Compared with the MAPE group, the NDI scores at 4 weeks were significantly lower in the DAPE group (*P* < 0.05).

#### 3.3.2. DAPE Improved Quality of Life More Than MAPE

Pain is often accompanied by depression and anxiety and decreased social function. Therefore, we used the SF-36 quality of life questionnaire to show the changes from baseline in patient-reported quality of life.

In the DAPE and MAPE groups, there were statistically significant increases in the weekly PCS and MCS scores after 1, 2, and 3 weeks of treatment compared to baseline values (Table 4). Compared with the SAE group, the PCS scores were significantly higher in the DAPE and MAPE groups after 2 weeks (*P* < 0.001 for both) and 3 weeks of treatment (95% CI, 11.0 to 27.1,* P* < 0.001 for DAPE; 95% CI, 1.9 to 18.1,* P* < 0.05 for MAPE). The PCS scores were significantly higher in the DAPE group compared with the MAPE group after 3 weeks of treatment (*P* < 0.05). Compared with the SAE group, the MCS scores were significantly higher in the DAPE and MAPE groups after 2 weeks (*P* < 0.001 for both ) and 3 weeks (95% CI, 12.3 to 26.9,* P* < 0.001 for DAPE; 95% CI, 11.1 to 25.7,* P* < 0.001 for MAPE) of treatment. Compared with baseline values, both the PCS and MCS scores at 10 weeks, the last follow-up time point, were significantly higher in the DAPE group (*P* < 0.001 for both), indicating improved physical and mental health by DAPE.

### 3.4. Safety

No serious adverse effects such as inflammatory granuloma or abnormal response were reported during the 3 weeks' treatment or the follow-up period (4 weeks and 10 weeks after the first treatment). Only one patient from DAPE group and two patients from the MAPE group complained of a tingling sensation after insertion in the acupoints located on the neck. These expected, non-serious adverse effects were self-limited, and no permanent injuries occurred. All adverse effects were reported as mild or moderate, and none required special medical interventions. The five patients fully recovered from the adverse effects and did not withdraw from the trial.

## 4. Discussion

To the best of our knowledge, this is the first randomized controlled trial investigating the effectiveness of APE in the treatment of CSR, especially neck and shoulder pain. The present study suggests that 3 weeks of APE treatment gave significant clinically relevant benefits in alleviating CSR-induced pain and improving clinical symptoms and quality of life, and we found better therapeutic effects when embedding the sutures in the deep layer (the* multifidus* muscle layer) compared with the middle layer (the* semispinalis capitis* muscle layer).

The analgesic effects and symptom improvements in response to APE might occur through relieving nerve compression and eliminating inflammatory edema in CSR patients. From the perspective of modern medicine, CSR develops from secondary inflammatory damage due to stimulation and hyperplasia of the nerve root by cervical disc herniation or joint hypertrophy, which causes shoulder and arm pain and numbness [1]. Thus, relieving nerve compression and eliminating inflammatory edema is critical for CSR treatment.

The present study sought to determine the efficacy of the novel material PGLA for clinical use in AE. PGLA and PGA are two synthetic absorbable sutures that have become widely available. Both suture types elicited minimal tissue response. Histologic examination showed that the PGLA sutures were absorbed by 90 days [14], which might be the underlying mechanism of persistent analgesic effects observed in the follow-up period (4 weeks and 10 weeks after the first treatment) in the DAPE group. In a previous report, a delayed foreign body granuloma associated with PGA sutures was diagnosed after 10 months following surgery for resection of a cerebral glioblastoma [21]. This adverse effect might have been because longer lengths of PGA were needed to close the incision. PGLA has good biocompatibility, and no adverse reactions have been reported. Moreover, only short lengths of PGLA (1 cm) were injected into each acupoint. Therefore, in the present study, no severe adverse effects were observed during either the treatment or follow-up periods. PGLA is hypothesized to stimulate the acupoints mainly through physical stimulation similar to the needle used in manual acupuncture treatment, but showing longer duration of effects due to the slow absorption of the material. The embedded sutures might subsequently activate the immune system and thus relieve pain and clinical symptoms in CSR patients.

We adopted the simplified acupoint selection principle that is often used in the clinic, and we selected the neck Jiaji and Dazhui acupoints but not the conventional acupoints in the distal limbs. According to the anatomical structure, Jiaji acupoint acupuncture can directly reach the nerve root, the intervertebral foramen, and the degenerative intervertebral disc and can improve the blood circulation in the affected area, promote the local metabolism, eliminate inflammatory mediators, and reduce neuroinflammatory reactions and edema [1, 10] and thus eliminate the neck pain and limb numbness. Jiaji AE is a minimally invasive and effective treatment for CSR and is easy to apply.

The relationship between the effectiveness of AE and the depth of the needle piercing for CSR treatment has been a matter of debate. The conflicting results in previous studies might be due to the indefinite depth and location of the acupuncture needle under the acupoints. Our previous research showed that ultrasound could clearly show the anatomical structure and tissue level of the cervical Jiaji acupoints, thus allowing for the visualization of the needling and the implantation [10].

The present study found that VAS scores after 3 weeks of treatment were significantly lower in the DAPE group than in the MAPE group, indicating that the effects of DAPE were significantly better than those of MAPE. According to our preliminary experiment, MAPE showed a greater decrease in VAS scores compared with DAPE; therefore, for sample size calculation, we used 3 and 2 as the mean expected decreases in VAS scores for the MAPE and DAPE groups, respectively. This difference might be because of the small sample size during the preliminary observations. The acupuncturist could feel Deqi at many muscle layers, while the patients felt the most significant distending pain only in the* multifidus* muscle layer, which might underlie the better therapeutic effects we observed for DAPE compared with MAPE.

The present study has some strengths and limitations. Strengths of this study include a randomized-controlled design and high participation rates. The trial had a good quality assurance plan to guarantee the quality of the data. Nevertheless, the study has some limitations. First, the participants were recruited primarily through outpatient departments and might not be representative of all patients with CSR. Second, like other clinical investigations, outcome measures such as VAS and NDI rely heavily on self-reporting, and their positive outcomes are likely to be overestimated. Third, our study used a semistandard prescription with fewer acupoints stimulated, and because we focused on efficacy in the present study, we did not use personalized treatment planning that is based on the physicians' experiences, which might cause performance bias. Further studies with a larger sample size and more controls are warranted.

## 5. Conclusion

As a traditional alternative therapy, APE is a promising method and a good choice for the treatment of pain and disability due to CSR. Additional studies with long-term clinical trials and animal experiments investigating the underlying mechanisms are eagerly expected for a better understanding of the application of APE.

## Figures and Tables

**Table 1:** Baseline characteristics of the 102 patients included in the intention-to-treat analysis.

Characteristics	SAE (n = 34)	MAPE (n = 34)	DAPE (n = 34)	*P*	All Patients (n = 102)
Women, n (%)	24 (70.6)	25 (73.5)	23 (67.6)	0.9	72 (70.6)
Age (years), mean ± SD	51.0 ± 12.9	54.0 ± 11.0	51.4 ± 13.9	0.6	52.1 ± 12.6
Duration of illness (months), mean ± SD	68.5 ± 47.3	56.5 ± 36.6	77.8 ± 64.1	0.4	67.6 ± 50.9

SAE, sham acupoint embedding group; MAPE, middle-layer (the *semispinalis capitis* muscle layer) PGLA embedding group; DAPE, deep-layer (the *multifidus* muscle layer) PGLA embedding group.

**Table 2:** Primary outcome measures.

Outcome Measure	SAE (n = 34)	MAPE (n = 34)	DAPE (n = 34)	Tukey's comparison					
				MAPE vs. SAE		DAPE vs. SAE		DAPE vs. MAPE	
				Diff. (95% CI)	*P*	Diff. (95% CI)	*P*	Diff. (95% CI)	*P*
Visual Analog Scale (VAS), Mean ± SD									
Baseline	6.1 ± 0.6	5.5 ± 1.0	5.4 ± 1.8	−0.6 (−1.2 to 0.0)	>.05	−0.6 (−1.2 to 0.0)	>.05	−0.0 (−0.7 to 0.6)	>.05
T 1w	5.8 ± 0.9	4.2 ± 1.2	3.7 ± 1.4	−1.5 (−2.2 to −0.9)	<.001	−2.1 (−2.7 to −1.4)	<.001	−0.5 (−1.2 to 0.1)	>.05
T 2w	6.2 ± 1.1	3.0 ± 1.0	2.5 ± 1.5	−3.3 (−3.9 to −2.6)	<.001	−3.7 (−4.4 to −3.1)	<.001	−0.4 (−1.1 to 0.2)	>.05
T 3w	5.9 ± 0.8	2.5 ± 1.2	1.6 ± 0.7	−3.4 (−4.0 to −2.7)	<.001	−4.2 (−4.9 to −3.6)	<.001	−0.9 (−1.5 to −0.3)	<.01
F 4w	5.8 ± 0.8	2.9 ± 1.1	2.4 ± 1.5	−2.9 (−3.5 to −2.2)	<.001	−3.4 (−4.0 to −2.8)	<.001	−0.5 (−1.2 to 0.1)	>.05
F 10w	6.0 ± 0.9	2.2 ± 0.7	2.2 ± 0.5	−3.8 (−4.4 to −3.2)	<.001	−3.8 (−4.4 to −3.2)	<.001	0.0 (−0.6 to 0.6)	>.05
P(t)	0.109	<.001	<.001	NA					

SAE, sham acupoint embedding group; MAPE, middle-layer (the *semispinalis capitis* muscle layer) PGLA embedding group; DAPE, deep-layer (the *multifidus* muscle layer) PGLA embedding group; CI, confidence interval; NA, not applicable. 1w, 1 week; 2w, 2 weeks; and 3w, 3 weeks; 4w, 4 weeks; 10w, 10 weeks; T, treatment; F, follow-up. P(t) refers to the p-values between different treatment time points.

**Table 3:** Secondary outcome measures (the clinical symptom and function assessment).

Outcome Measure	SAE (n = 34)	MAPE (n = 34)	DAPE (n = 34)	Tukey's Comparison					
				MAPE vs. SAE		DAPE vs. SAE		DAPE vs. MAPE	
				Diff. (95% CI)	*P*	Diff. (95% CI)	*P*	Diff. (95% CI)	*P*
**Yasuhisa Tanaka 20 (YT-20), Mean ± SD**									
Baseline	13.1 ± 2.6	12.1 ± 3.4	12.6 ± 3.2	−1.0 (−3.1 to 1.0)	>.05	−0.9 (−2.9 to 1.0)	>.05	0.1 (−1.8 to 2.0)	>.05
T 1w	13.6 ± 2.7	13.0 ± 3.4	13.5 ± 3.2	−0.6 (−2.7 to 1.4)	>.05	−0.1 (−2.1 to 1.8)	>.05	0.5 (−1.4 to 2.4)	>.05
T 2w	13.5 ± 2.5	14.9 ± 2.2	15.3 ± 2.8	1.6 (−0.5 to 3.6)	>.05	1.7 (−0.2 to 3.7)	<.05	0.2 (−1.7 to 2.1)	>.05
T 3w	13.2 ± 2.6	17.5 ± 1.6	17.4 ± 1.9	4.3 (2.3 to 6.3)	<.001	4.2 (2.2 to 6.1)	<.001	−0.1 (−2.0 to 1.8)	>.05
F 4w	13.4 ± 2.7	13.9 ± 2.7	12.5 ± 3.3	−0.2 (−2.1 to 1.6)	>.05	−2.4 (−4.2 to -0.6)	>.05	−2.2 (−3.9 to −0.4)	>.05
F 10w	13.5 ± 2.5	14.6 ± 2.0	14.7 ± 3.0	1.1 (−0.5 to 2.6)	>.05	0.4 (−1.4 to 2.2)	>.05	−2.2 (−3.9 to −0.4)	>.05
P(t)	0.053	<.001	<.001	NA					
**Neck Deficiency Index (NDI), Mean ± SD**									
Baseline	40.7 ± 4.5	39.3 ± 6.7	39.8 ± 7.0	−1.5 (−4.9 to 2.0)	>.05	−0.9 (−4.4 to 2.6)	>.05	−4.4 (−11.9 to 3.1)	>.05
T 1w	40.0 ± 4.8	37.5 ± 8.4	35.5 ± 6.2	−2.4 (−5.9 to 1.0)	>.05	−4.5 (−8.0 to −1.1)	<.01	−5.4 (−13.0 to 2.1)	>.05
T 2w	40.5 ± 4.8	39.1 ± 7.4	35.8 ± 7.1	−1.4 (−4.9 to 2.0)	>.05	−4.7 (−8.2 to −1.3)	<.01	−5.9 (−13.5 to 1.6)	>.05
T 3w	39.9 ± 4.8	34.4 ± 6.7	31.5 ± 5.6	−5.5 (−9.0 to −2.0)	<.001	−8.4 (−11.9 to −5.0)	<.001	−4.7 (−12.2 to 2.9)	>.05
F 4w	39.3 ± 4.4	35.4 ± 5.7	31.9 ± 5.9	−4.0 (−7.4 to −0.5)	<.05	−7.4 (−10.9 to -4.0)	<.001	−5.0 (−12.5 to 2.5)	<.05
F 10w	39.7 ± 4.8	30.1 ± 6.3	27.6 ± 6.2	−9.6 (−13.1 to −6.2)	<.001	−12.1 (−15.5 to −8.6)	<.001	−4.0 (−11.5 to 3.6)	>.05
P(t)	0.119	<.001	<.001	NA					

SAE, sham acupoint embedding group; MAPE, middle-layer (the *semispinalis capitis* muscle layer) PGLA embedding group; DAPE, deep-layer (the *multifidus* muscle layer) PGLA embedding group; CI, confidence interval; NA, not applicable. 1w, 1 week; 2w, 2 weeks; 3w, 3 weeks; 4w, 4 weeks; 10w, 10 weeks; T, treatment; F, follow-up. P(t) refers to the p-values between different treatment time points.

**Table 4:** Secondary outcome measures (quality of life assessment).

Outcome Measure	SAE (n = 34)	MAPE (n = 34)	DAPE (n = 34)	Tukey's Comparison					
				MAPE vs. SAE		DAPE vs. SAE		DAPE vs. MAPE	
				Diff. (95% CI)	*P*	Diff. (95% CI)	*P*	Diff. (95% CI)	*P*
**The Physical Component Summary (PCS) of the SF36 health questionnaire, Mean ± SD**									
Baseline	56.4 ± 13.8	54.4 ± 13.8	57.4 ± 19.8	−2.0 (−10.1 to 6.1)	>.05	0.9 (−7.1 to 9.0)	>.05	3.9 (−5.1 to 12.9)	>.05
T 1w	56.9 ± 14.6	58.0 ± 14.4	59.8 ± 19.2	1.1 (−7.0 to 9.2)	>.05	2.9 (−5.2 to 10.9)	>.05	2.8 (−6.3 to 11.8)	>.05
T 2w	56.3 ± 14.5	68.9 ± 11.8	69.3 ± 13.8	12.6 (4.5 to 20.7)	<.001	13.0 (4.9 to 21.1)	<.001	1.6 (−7.5 to 10.6)	>.05
T 3w	56.7 ± 13.56	66.7 ± 11.4	75.7 ± 12.7	10.0 (1.9 to 18.1)	<.05	19.0 (11.0 to 27.1)	<.001	10.1 (1.0 to 19.1)	<.05
F 4w	57.0 ± 14.5	69.9 ± 11.8	69.0 ± 15.4	12.9 (4.8 to 21.0)	<.001	11.9 (3.9 to 20.0)	<.01	0.4 (−7.4 to 8.2)	>.05
F 10w	57.7 ± 13.9	75.2 ± 9.3	74.2 ± 13.1	17.5 (9.4 to 25.6)	<.001	16.5 (8.4 to 24.6)	<.001	−0.4 (−8.2 to 7.3)	>.05
P(t)	0.129	<.001	<.001	NA					
**The Mental Component Summary (MCS) of the SF36 health questionnaire, Mean ± SD**									
Baseline	55.7 ± 13.3	54.8 ± 14.6	56.9 ± 15.9	−0.9 (−8.2 to 6.4)	>.05	1.2 (−6.1 to 8.5)	>.05	2.1 (−5.2 to 9.4)	>.05
T 1w	57.9 ± 11.6	61.1 ± 17.4	63.4 ± 18.0	3.1 (−4.2 to 10.5)	>.05	5.4 (−1.9 to 12.8)	>.05	2.3 (−5.0 to 9.6)	>.05
T 2w	57.0 ± 11.6	76.6 ± 11.3	77.8 ± 11.5	19.6 (12.3 to 26.9)	<.001	20.8 (13.5 to 28.1)	<.001	1.1 (−6.2 to 8.5)	>.05
T 3w	57.3 ± 11.0	75.6 ± 8.9	76.9 ± 10.1	18.4 (11.1 to 25.7)	<.001	19.6 (12.3 to 26.9)	<.001	1.2 (−6.1 to 8.5)	>.05
F 4w	58.2 ± 12.2	63.3 ± 13.5	63.1 ± 10.9	5.0 (−2.3 to 12.4)	>.05	4.9 (−2.4 to 12.2)	>.05	−0.1 (−7.4 to 7.2)	>.05
F10w	58.7± 11.7	62.8 ± 11.1	64.5 ± 12.1	4.1 (−3.2 to 11.4)	>.05	5.8 (−1.5 to 13.1)	>.05	1.7 (−5.6 to 9.0)	>.05
P(t)	0.058	<.001	<.001	NA					

SAE, sham acupoint embedding group; MAPE, middle-layer (the *semispinalis capitis* muscle layer) PGLA embedding group; DAPE, deep-layer (the *multifidus* muscle layer) PGLA embedding group; CI, confidence interval; NA, not applicable. 1w, 1 week; 2w, 2 weeks; 3w, 3 weeks; 4w, 4 weeks; 10w, 10 weeks; T, treatment; F, follow-up. P(t) refers to the p-values between different treatment time points.

## Data Availability

The data used to support the findings of this study are available from the corresponding author upon request.
